# Causal attribution in forensic psychological injury: legal causation, validity assessment, and expert reasoning

**DOI:** 10.3389/fpsyg.2026.1877102

**Published:** 2026-07-09

**Authors:** Juliane Tortes Saint-Jammes

**Affiliations:** CAMEA, Léognan, France

**Keywords:** causal attribution, expert testimony, forensic psychology, imputability, legal causation, malingering, performance and symptom validity, psychological injury

## Abstract

Forensic psychological injury evaluations often require an opinion on whether post-event symptoms are attributable to a specific accident, assault, workplace exposure, institutional event, or alleged abuse. Such opinions cannot rest on temporal sequence, clinical plausibility, or the presence of a trauma-related diagnosis alone. This conceptual analysis argues that defensible causal attribution requires coordination of three elements that are often handled separately: legal causation concepts, psychological causal reasoning, and validity assessment. The article distinguishes event occurrence, psychological injury, functional impairment, and imputability of injury to the event. It then discusses but-for causation, material contribution, proximate or legal causation in common-law settings, and the broader problem of imputability in civil-law contexts. The article further argues that performance validity tests, symptom validity tests, and broader response-validity analysis are not ancillary psychometric issues but conditions that shape the permissible strength of causal conclusions. A revised framework is proposed that distinguishes direct causation, material contributory causation, indirect causal pathways, and insufficient basis for attribution, while treating precipitating, exacerbating, accelerating, cumulative, and perpetuating effects as modifiers. The framework is positioned relative to prior work on psychological injury, malingering, and forensic evaluation, and is translated into a practical reporting sequence, a short vignette, and calibrated wording for expert reports.

## 1 Introduction

Courts, compensation systems, insurers, child-protection authorities, and other legal or quasi-legal decision-makers frequently ask psychologists to determine whether a person’s psychological condition can be attributed to a specific event. The practical question is usually formulated in legal language: did the accident, assault, workplace exposure, medical incident, institutional response, or alleged abuse cause the psychological injury? In civil matters, the answer may influence compensation, disability determinations, apportionment, or settlement. In criminal and protective contexts, it may affect how psychological harm is understood, how risk is formulated, and how testimony is weighed. The expert is therefore asked to translate incomplete psychological evidence into a legally usable causal opinion.

The task is usually more difficult than the referral wording suggests. Symptoms are often reported months or years after the event. Pre-event mental health data may be missing, and current functioning may reflect prior psychopathology, developmental adversity, medical illness, pain, medication, sleep disturbance, unemployment, family conflict, litigation stress, or the social consequences of the event. Trauma-informed frameworks have improved the recognition and treatment of trauma-related distress (Briere and Scott, 2014), but they can be misapplied when their clinical logic is transferred to forensic reasoning without methodological safeguards. A diagnosis describes a clinical pattern; it does not, by itself, establish why that pattern developed in a particular person.

The difficulty is also legal. Legal systems distinguish factual causation from normative or legal responsibility, and they require expert evidence to be grounded in sufficient information, reliable methods, and careful application to the facts of the case. In psychological injury work, those requirements cannot be separated from data quality. When symptom reports, test scores, or functional claims are invalid, inconsistent, or insufficiently corroborated, any causal opinion built on them must be weakened accordingly.

This article is a conceptual analysis, not an empirical study or a systematic review. It integrates legal causation concepts, forensic validity assessment, and psychological causal reasoning into a practical framework for expert reporting. Its novelty is not the claim that psychological causation is multifactorial; that is already well recognised. The contribution is the explicit linkage between the strength of a causal opinion and the validity of the data on which it rests, the separation of primary causal categories from causal modifiers and evidentiary insufficiency, and the formulation of report language that can be adapted across common-law and civil-law settings.

The argument is organised as follows. The next section defines the method and scope of the analysis. The article then distinguishes legal causation from psychological explanation, with attention to common-law concepts and to civil-law imputability. It subsequently examines the boundary between clinical plausibility and forensic attribution, the role of PVTs and SVTs, the proposed causal framework, and the reporting implications of the model.

## 2 Method and scope

This article uses an integrative conceptual approach. It does not report primary data and does not claim the exhaustive retrieval standards of a systematic review. The aim is narrower: to articulate a coherent framework for causal reasoning in forensic psychological injury by bringing together literature on expert testimony and psychological injury, performance and symptom validity, trauma-related diagnosis, and causation in legally contested settings.

The sources were selected through targeted database and citation searches rather than through a preregistered review protocol. Searches combined terms such as causation, causal attribution, psychological injury, forensic psychology, expert testimony, malingering, performance validity, symptom validity, PTSD, and legal causation. Retrieval was supplemented by citation chaining from key consensus statements, review articles, and publications in psychological injury and forensic neuropsychology. Major common-law admissibility cases are cited to clarify the reliability logic that often structures expert evidence; they are not treated as a complete comparative-law analysis.

Accordingly, the article should be read as a conceptual and methodological proposal. Statements about practice refer to patterns described in the literature and to the author’s forensic experience in France; they are not presented as empirical prevalence estimates. The limitations of this approach are addressed in the discussion.

## 3 Legal causation and the proper role of psychological expertise

Forensic psychologists do not decide legal responsibility. Their role is to assist the trier of fact by describing psychological injury, functional impairment, the validity of the data, and the extent to which the available evidence supports a causal link between the event at issue and the claimed condition (American Psychological Association, 2013; Melton et al., 2017). The distinction matters because legal causation is not the same as clinical explanation. Clinical formulation may be broad, narrative, and therapeutic. Legal causation is constrained by the referral question, evidentiary rules, and the applicable standard of proof.

### 3.1 Common-law frameworks

Most common-law systems distinguish factual causation from legal or proximate causation. Factual causation is often framed as a but-for question: would the injury have occurred but for the event at issue? In psychological injury cases, the answer is rarely straightforward. A claimant may present with pre-existing vulnerabilities, prior trauma, chronic anxiety, personality features, medical comorbidities, or concurrent stressors. The expert must therefore ask not only whether symptoms followed the event, but whether the evidence supports a reasoned conclusion that the event changed the onset, severity, course, or functional consequences of the condition. Comparable caution is emphasised in the forensic medicine literature on causal inference (Meilia et al., 2020).

Multiple causation requires particular care. Where several factors plausibly contributed to the injury, the relevant question may be whether the event was a material contributor rather than the sole cause. A psychologically significant contribution is not necessarily a legally material contribution, and a legally material contribution does not require psychological exclusivity. The expert’s task is to identify the empirical and clinical basis for any asserted contribution, describe competing explanations, and avoid converting plausibility into certainty.

Admissibility and reliability standards reinforce this methodological discipline. Under Daubert and related case law in the United States (Daubert v. Merrell Dow Pharmaceuticals, Inc., 1993; General Electric Co. v. Joiner, 1997; Kumho Tire Co. v. Carmichael, 1999), expert evidence is evaluated with reference to features such as testability, peer review, error rates, standards controlling the technique, and general acceptance. Even when another jurisdiction applies different admissibility rules, the underlying scientific expectation is similar: the expert must show the method by which the conclusion was reached. In psychological injury evaluations, this requires attention to the quality of the data, the reliability and validity of instruments used, the relevance of base rates, and the limits of individual inference drawn from group-level research (Faigman et al., 2014).

### 3.2 Civil-law frameworks and imputability

Civil-law systems often frame the expert question less in terms of common-law proximate cause and more in terms of whether a given impairment is imputable to the event under examination. The doctrinal structure varies across jurisdictions, and the present article does not attempt a full comparative legal account. For psychological experts, however, the practical task is similar: to describe whether the event appears to have made a demonstrable difference to the person’s mental state or functioning, while distinguishing pre-existing conditions, concurrent stressors, and evidentiary uncertainty.

In the French medico-legal context, for example, experts are often asked to distinguish an anterior state from post-event impairment and to give an opinion on imputability rather than on legal liability. That task requires attention to baseline functioning, temporal course, plausible mechanism, collateral documentation, and the validity of symptom and performance data. A conclusion that an injury is psychologically imputable to an event is therefore not merely a statement that the person reports symptoms after the event; it is an evidentiary judgement about the most defensible link between the event and the impairment.

Across jurisdictions, causal language needs calibration. Saying that symptoms are consistent with trauma is not the same as saying that the event caused the condition. Saying that an event contributed to distress is not the same as saying that it was a legally material cause of impairment. The framework proposed here treats expert causation as a structured opinion about psychological imputability, intended to inform rather than replace the court’s legal judgement.

## 4 From clinical plausibility to forensic attribution

A recurrent error in psychological injury evaluation is the compression of three questions into one: whether the event occurred, whether the person has symptoms or a disorder, and whether those symptoms are attributable to the event. Clinical practice may blend these questions because treatment is oriented toward meaning, alliance, and symptom reduction. Forensic practice must keep them separate. Symptoms do not prove event occurrence, and event occurrence does not automatically attribute all later symptoms to that event.

Temporal proximity is a weak causal criterion when used alone. Symptoms can emerge after an event because of the event, but also because of pre-existing conditions, natural fluctuation, medical problems, pain, sleep disruption, relationship deterioration, unemployment, financial stress, or the psychological burden of litigation itself. Conversely, symptoms may appear delayed because avoidance, dissociation, social support, or institutional context changed over time. The relevant forensic question is not whether a temporal narrative is coherent, but whether the totality of evidence differentially supports one causal explanation over plausible alternatives.

PTSD illustrates the risk of diagnostic reification. DSM diagnostic criteria include exposure and symptom criteria, but a PTSD diagnosis does not prove that a particular legal event is the unique or primary cause of the condition (McNally, 2003; Rosen and Lilienfeld, 2008). Trauma exposure is common (Kessler et al., 2017), PTSD prevalence varies by population and diagnostic rule (Hoge et al., 2014), and many symptoms—sleep disturbance, irritability, concentration problems, affective instability, somatic tension, and avoidance—are transdiagnostic. Diagnosis can support clinical description and treatment planning; it cannot substitute for forensic causal analysis (Zoellner et al., 2013).

Group-to-individual inference is another source of error. Epidemiological evidence can show that certain events increase the risk of PTSD, depression, anxiety, pain-related disability, or occupational impairment. It cannot, by itself, determine causation in the individual case (Faigman et al., 2014). The expert must connect general evidence to case-specific indicators: pre-event baseline, symptom onset and course, medical and psychological history, collateral records, functional change, mechanism of injury, consistency across sources, response validity, and the presence or absence of credible alternative explanations.

The standard of reasoning is therefore comparative rather than narrative. The expert should ask which causal model best accounts for the evidence and which observations would be expected if each model were true. A direct causal model predicts a meaningful discontinuity after the event, a plausible mechanism linking the event to symptoms, limited competing causes, and valid data. A contributory model predicts a more complex interaction amongst the event, vulnerabilities, and co-occurring stressors. An indeterminate conclusion is warranted when the data do not permit a reasoned preference amongst competing explanations.

## 5 Validity assessment as a precondition for causal reasoning

In forensic settings, validity assessment is neither an accusation nor a peripheral technical issue. It is part of the evidentiary foundation of the opinion. Psychological injury evaluations often rely on self-report, psychometric profiles, reported cognitive or somatic symptoms, and claimed functional limitations. If those data are invalid, exaggerated, inconsistent, or insufficiently tested, causal attribution becomes unstable. The problem is broader than intentional deception: low-quality data may reflect feigning, gross exaggeration, misunderstanding, poor effort, psychiatric disorganisation, pain, fatigue, cultural or linguistic factors, secondary gain, or the demand characteristics of litigation (Bass and Halligan, 2014; Frueh et al., 2000; Rogers and Bender, 2018).

PVTs and SVTs address different but related domains. Performance validity tests evaluate whether performance on cognitive or performance-based tasks is interpretable. Symptom validity tests evaluate whether self-reported symptoms are credible, internally consistent, and not grossly exaggerated relative to empirically established patterns (Larrabee, 2012; Heilbronner et al., 2009). Neither class of measures is a lie detector. Their function is to identify data quality problems that affect the interpretation of test results, diagnostic profiles, and causal conclusions. A passed validity screen does not prove symptom genuineness; a failed validity measure does not by itself prove malingering. Both results must be interpreted in a multimethod framework (Bush et al., 2014; Sherman et al., 2020).

This distinction has direct causal significance. If performance validity is poor, cognitive test results cannot be treated as reliable evidence of cognitive injury caused by the event. If symptom validity measures indicate extreme overreporting or a profile inconsistent with known clinical patterns, the symptom profile cannot be used uncritically to infer the severity, mechanism, or temporal origin of impairment. In such cases, the whole causal analysis must be qualified. The expert may still describe observed distress, contextual stressors, or functional difficulties, but should avoid strong attributional conclusions unless independent evidence — medical records, treatment history, occupational data, collateral observations — provides sufficient support.

Validity analysis should be proportional to the referral question and the evidentiary stakes. In high-stakes compensation, disability, criminal, or custody-related evaluations, a bare clinical interview is rarely sufficient (Merten et al., 2022). The expert should consider multiple sources of validity information: standalone PVTs, embedded performance indicators, SVTs or overreporting scales, consistency across records, collateral observations, behavioural data, treatment history, medical evidence, and functional documentation. The number and type of validity measures should be selected according to the domains being assessed, the nature of claimed impairment, the person’s abilities and language, and the known limitations of the instruments (American Educational Research Association et al., 2014).

Base rates and error rates are essential. The meaning of a validity failure depends partly on context, population, instrument, cutoff, number of tests administered, and the consequences of false-positive and false-negative errors (Mittenberg et al., 2002; Slick et al., 1999). The expert should avoid simplistic rules such as treating any single validity failure as malingering or treating the absence of validity failure as proof of accurate reporting. Where validity findings are mixed, the report should specify which domains remain interpretable and which do not. For causal attribution, the core question is whether the data supporting the causal chain are sufficiently valid to support the level of opinion offered.

A practical rule follows: the causal opinion should not be stronger than the evidence on which it depends. If the causal claim rests mainly on self-report and the self-report profile is invalid, strong attribution is not warranted. If independent records show a clear pre-to-post functional discontinuity, treatment change, work loss, behavioural change, or contemporaneous symptoms, an opinion of contribution may still be possible, but the validity limitations must be stated directly.

## 6 Revised causal framework

### 6.1 Positioning against existing frameworks

Several prior frameworks have addressed causal attribution in psychological injury. Young (2015) distinguished several causal categories applicable to both criminal forensic and civil disability contexts, including direct and contributory forms of causation as well as precipitating, exacerbating, and perpetuating influences. Foote et al. (2020) synthesised legal, professional, and ethical considerations for civil forensic evaluation in psychological injury. In the neuropsychological literature, Slick et al. (1999), later updated by Sherman et al. (2020), proposed multidimensional criteria for identifying malingered neurocognitive dysfunction. The American Academy of Clinical Neuropsychology consensus statement (Heilbronner et al., 2009) and the ASAPIL official position (Bush et al., 2014) established validity assessment as a standard-of-care expectation in forensic neuropsychological practice. European work (Merten et al., 2022) has extended these standards to civil-law jurisdictions.

The present framework extends these contributions in three ways. First, it treats validity assessment as a constraint on causal interpretation, not as a separate psychometric appendix. Second, it separates primary causal categories (direct, material contributory, indirect, insufficient basis) from temporal or functional modifiers (precipitating, exacerbating, accelerating, cumulative, perpetuating). Third, it uses language that can be adapted to common-law and civil-law contexts, including cases in which the operative question is imputability rather than proximate cause. Table 1 summarises the distinctions.

**Table 1:** Positioning of the proposed framework against prior approaches.

Prior framework	Main contribution	Point of extension in the present framework
Young (2015)	Distinguishes causal categories across criminal forensic and civil disability contexts, including direct, contributory, precipitating, exacerbating, and perpetuating forms.	Separates primary causal categories from temporal/functional modifiers; ties strength of opinion explicitly to data validity.
Foote et al. (2020)	Integrates legal, professional, and ethical considerations for civil forensic evaluation in psychological injury.	Adds an explicit reporting grammar calibrated across jurisdictions, including civil-law imputability.
Slick et al. (1999) and Sherman et al. (2020)	Multidimensional criteria for malingered neurocognitive dysfunction, focused on detection of feigning.	Generalises from detection of feigning to the broader problem of how validity findings constrain causal attribution.
Heilbronner et al. (2009), Bush et al. (2014), and Merten et al. (2022)	Establish validity assessment as standard of care in forensic neuropsychology (US and European contexts).	Integrates validity standards into causal reasoning as a precondition, not a parallel track.

### 6.2 Primary causal categories

Expert reports should not mix causal types, temporal descriptors, and evidentiary conclusions. The framework therefore distinguishes four primary conclusions from causal modifiers.

Direct causation refers to cases in which the event at issue is the most plausible and materially sufficient explanation for the onset of the psychological injury. This conclusion requires a documented pre-event baseline, a meaningful post-event discontinuity, a plausible psychological mechanism, valid data, and limited alternative explanations. In practice, direct causation is a strong opinion and should be used sparingly in complex psychological injury cases.

Material contributory causation refers to cases in which the event at issue is one important causal factor amongst others. It may interact with pre-existing vulnerabilities, prior trauma, ongoing pain, employment disruption, family conflict, or institutional stress. The key question is not whether other factors existed, but whether the event made a material difference to the onset, severity, persistence, or functional consequences of the injury. This category is often the most realistic forensic conclusion in psychological injury evaluations involving multiple stressors.

Indirect causal pathways refer to cases in which the event affects mental health through mediating mechanisms rather than through immediate psychological trauma alone. Examples include loss of employment, prolonged litigation, social isolation, financial deterioration, medical treatment complications, institutional betrayal, or the consequences of protective removal. These pathways can still be material if the mediating chain is sufficiently documented and not merely speculative.

Insufficient basis for attribution is warranted when the evidence supports compatibility or temporal association but not a reasoned causal conclusion. This may occur because baseline data are absent, alternative explanations are substantial, the symptom course is unclear, validity findings are problematic, or the alleged mechanism is weakly supported. This conclusion is not a failure of expertise; it is often the most scientifically defensible answer.

### 6.3 Modifiers

Modifiers can be attached to the primary categories when they are supported by the evidence. A factor may be precipitating if it triggers onset, exacerbating if it increases severity, accelerating if it speeds an already developing trajectory, cumulative if its effect depends on additive adversity, and perpetuating if it maintains symptoms after onset. These terms refine the causal opinion. They should not replace the primary causal category (Table 2).

**Table 2:** Revised causal categories for forensic psychological injury.

Primary conclusion	Core meaning	Minimum evidentiary requirements	Reporting cautions
Direct causation	The event is the most plausible and materially sufficient explanation for the injury.	Documented pre-event baseline; clear post-event discontinuity; plausible mechanism; valid data; weak alternative explanations.	Use sparingly in complex cases; avoid when multiple substantial causes are documented.
Material contributory causation	The event made a meaningful difference to onset, severity, persistence, or impairment amongst other factors.	Evidence that the event changed trajectory or functional outcome; explicit account of pre-existing and concurrent causes; interpretable validity findings.	Do not imply sole cause; distinguish psychological contribution from legal apportionment.
Indirect causal pathway	The event affected mental health through mediating consequences such as institutional, medical, social, or occupational disruption.	Documented chain linking event to mediator and mediator to symptoms or impairment; alternatives considered.	Specify whether the pathway is material or merely speculative.
Insufficient basis for attribution	The evidence supports compatibility or temporal association but not a reasoned causal conclusion.	Missing baseline, major alternative causes, invalid data, unclear trajectory, or weak mechanism.	State what is unknown and why stronger attribution is not justified.
Modifiers	Precipitating, exacerbating, accelerating, cumulative, and perpetuating effects describe how causes operate.	Evidence for timing, interaction, worsening, persistence, or cumulative load.	Use as qualifiers attached to a primary conclusion, not as stand-alone causal categories.

## 7 Methodological sequence, illustrative vignette, and reporting template

### 7.1 Methodological sequence

A structured causal opinion begins with the injury itself: symptoms, diagnosis when warranted, severity, duration, and functional impairment. The report should distinguish subjective distress from occupational, interpersonal, parenting, educational, or daily-life impairment, because legal relevance often turns on impairment rather than on diagnosis alone.

The next step is to define the causal question. The report should identify the event at issue, the relevant time window, and the type of causal relation being evaluated. A question about acute symptom triggering differs from a question about long-term disability; a question about PTSD differs from a question about depression, pain behaviour, or global functional decline.

Baseline and trajectory then need to be reconstructed from the available material: prior treatment records, employment or school data, medical information, collateral sources, and the examinee’s account. The key issue is whether the record supports a credible discontinuity, acceleration, or deterioration after the event, and whether that pattern fits the proposed mechanism.

Validity and data quality should be evaluated before the causal conclusion is stated. Relevant sources include PVT and SVT findings, response style, consistency across records, plausibility of symptom endorsement, behavioural observations, and possible incentives. The report should identify which data are interpretable, which are questionable, and which should not be used as the basis for causal inference.

Competing causal hypotheses should be compared explicitly. At a minimum, these usually include the event-at-issue hypothesis, pre-existing condition hypothesis, concurrent stressor hypothesis, cumulative adversity hypothesis, medical or pain-related hypothesis, litigation or institutional stress hypothesis, and insufficient-evidence hypothesis.

The conclusion should then use calibrated language. Compatibility, contribution, material contribution, direct causation, indirect pathway, and insufficient basis are not interchangeable. A useful opinion states the level of confidence and the reasons for any limits, rather than offering categorical language that the evidence cannot support.

### 7.2 Illustrative forensic vignette

Consider a claimant evaluated eighteen months after a motor vehicle accident. The claimant reports PTSD symptoms, cognitive fog, chronic pain, irritability, inability to work, and marked social withdrawal. The accident is documented, but emergency records indicate no loss of consciousness and no acute psychiatric evaluation. Pre-event records show intermittent depression, prior workplace conflict, and treatment for chronic insomnia. Post-event records show increased medical visits, physiotherapy, antidepressant adjustment, and later litigation-related stress. Collateral information confirms reduced work attendance after the accident but also indicates ongoing family conflict and financial strain.

A clinically plausible formulation might state that the accident explains the current psychological condition. A forensic formulation should be more constrained. The accident is temporally associated with worsening distress and functional disruption. The medical and occupational records support some post-event deterioration. However, the pre-existing depression and insomnia, chronic pain, family conflict, and litigation stress are competing contributors. If PVTs are passed but SVTs show elevated overreporting on somatic and psychiatric scales, cognitive test results may remain interpretable while symptom severity estimates require caution.

In this vignette, the most defensible opinion is likely to be material contributory causation rather than direct causation: the accident plausibly contributed to psychological deterioration and work disruption in interaction with pre-existing vulnerabilities and subsequent stressors. If validity findings were globally invalid, the opinion would need to shift toward compatibility or insufficient basis for attribution, unless independent records provided stronger support. The same facts therefore produce different causal conclusions depending on the quality of the data and the legal question being answered.

### 7.3 Reporting template and wording conventions

Forensic reports should make causal reasoning auditable. A minimal reporting template includes five components. First, identify the alleged event and the legal or referral question. Second, define the claimed injury and functional impairment. Third, state the evidentiary basis, including interviews, tests, records, collateral information, and validity findings. Fourth, compare competing causal hypotheses. Fifth, provide a calibrated conclusion with explicit limitations.

Wording should avoid equivocal phrases that courts may overinterpret. Consistent with should be reserved for compatibility, not causation. Contributed to should be used only when evidence supports a causal role amongst other factors. Materially contributed to should be reserved for cases in which the evidence supports that the event made a meaningful difference to onset, severity, persistence, or functional impairment. Directly caused should be reserved for cases with strong baseline, trajectory, mechanism, and validity evidence. Insufficient basis should be used when data quality, alternative causes, or missing information prevent a reasoned attribution.

Example language for compatibility: the reported symptoms are compatible with a post-event stress response, but the available evidence does not permit attribution to the event at issue rather than to other antecedent or concurrent factors. Example language for material contribution: the event at issue appears to have materially contributed to the current psychological condition, particularly by exacerbating pre-existing vulnerabilities and producing documented functional decline; however, the relative weight of each factor cannot be quantified with precision. Example language for insufficiency: given the absence of baseline data, multiple plausible alternative explanations, and validity limitations affecting the symptom profile, causal attribution to the event at issue cannot be established to a reasonable degree of forensic certainty.

This style of reporting does not weaken expert testimony. It clarifies it. A transparent opinion allows the court to decide how psychological evidence fits within the applicable legal standard, while reducing the risk that clinical sympathy, narrative coherence, or diagnostic labels will be mistaken for proof of legal causation (Table 3).

**Table 3:** Minimal reporting template for causal conclusions.

Report component	Question to answer	Recommended content
Injury and impairment	What is being attributed?	Symptoms, diagnosis if warranted, severity, duration, and concrete functional impairment.
Evidence base	What information supports the opinion?	Interview, records, psychometric data, collateral sources, behavioural observations, and limitations.
Validity	Are the data interpretable?	PVT/SVT results when relevant, response style, consistency, overreporting, and domain-specific interpretability.
Causal hypotheses	What explanations were compared?	Event-at-issue model, pre-existing conditions, concurrent stressors, medical or pain mechanisms, cumulative adversity, and insufficient-evidence hypothesis.
Causal conclusion	What level of attribution is justified?	Compatibility only, material contribution, direct causation, indirect pathway, or insufficient basis.
Uncertainty	What limits confidence?	Missing baseline, validity concerns, conflicting records, alternative causes, or unknown chronology.

## 8 Discussion, limitations, and conclusion

### 8.1 Implications for practice

The framework responds to a recurrent problem in forensic psychological injury: the pressure to transform complex, probabilistic, and incomplete psychological evidence into a binary causal answer. Event occurrence, symptom presence, diagnosis, impairment, validity, and causal attribution are distinct steps. Failure to separate them increases the risk of over-attribution, under-analysis of alternative causes, and legally misleading conclusions.

The main practical implication is that validity assessment belongs inside the causation analysis rather than beside it. Invalid symptom or performance data limit the causal inferences that can be drawn from them. Conversely, valid data do not establish causation by themselves; they only make causal analysis possible. This distinction is especially important in personal injury, disability, criminal, and family-law contexts, where external incentives, emotional stakes, and institutional pressures may affect presentation. It is also important in civil-law settings, where validity evidence may be less routinely requested than in adversarial common-law practice.

A second implication concerns the relation between law and psychology. Psychologists should not attempt to resolve legal responsibility, but they should understand the legal causal questions they are asked to inform. But-for causation, material contribution, proximate cause, and imputability do not map perfectly onto clinical explanation. Reports that ignore this mismatch risk being clinically rich but legally ambiguous. Reports that reduce psychological injury to legal formulas risk losing scientific nuance. The goal is a disciplined interface: psychological expertise expressed in language that is legally interpretable and methodologically transparent.

### 8.2 Limitations

The framework has clear limitations. It is a conceptual model, not a validated decision algorithm. It does not assign numerical probabilities to causal conclusions and does not replace jurisdiction-specific legal analysis. The integrative method used here is transparent but non-systematic; a systematic review of causal frameworks in forensic mental-health practice would be a useful next step. The choice of PVTs, SVTs, collateral records, and diagnostic instruments must be adapted to the referral question, population, language, culture, and available evidence. Some evaluations will lack sufficient records or will involve urgent protective decisions in which full testing is not feasible; in those contexts, the expert should state the limits rather than inflate confidence. Finally, the framework is more strongly grounded in common-law scholarship than in civil-law doctrinal sources, so further comparative legal-psychological work is needed.

### 8.3 Directions for future work

Future work should examine how experts currently communicate causal reasoning in psychological injury reports, whether structured causal templates improve clarity for judges and attorneys, and how validity findings alter causal conclusions in real-world cases. Vignette studies could test whether legal decision-makers distinguish compatibility, contribution, material contribution, direct causation, and insufficient basis when experts use calibrated wording. Comparative studies across jurisdictions could clarify how imputability reasoning interacts with psychometric validity evidence.

## 9 Conclusion

Causal attribution in forensic psychological injury is not a direct translation of clinical formulation into legal language. It is a constrained inference requiring separation of event occurrence, injury, functional impairment, validity, and imputability. Trauma-informed reasoning is clinically valuable, but it becomes forensic overreach when exposure plus symptoms is treated as proof of causation. A defensible expert opinion should be no stronger than the quality of the data, the validity of the presentation, and the evidence supporting the proposed mechanism. Direct causation, material contributory causation, indirect causal pathways, and insufficient basis for attribution provide a clearer hierarchy than mixed lists that combine causes, modifiers, and evidentiary conclusions. Methodological restraint is not a weakness of expert testimony; it is the condition under which psychological evidence remains useful, ethical, and scientifically credible in legal settings.
